# Perceived Stigma in People Living With HIV in Qom 

**Published:** 2017-12

**Authors:** Davoud Pourmarzi, Ashraf Khoramirad, Mina Gaeeni

**Affiliations:** 1 Reproductive Health Research Center, Guilan University of Medical Sciences, Rasht, Iran; 2 Faculty of Nursing and Midwifery, Qom University of Medical Sciences, Qom, Iran

**Keywords:** HIV, Social Stigma, Social Support, Rural Population, Unemployment

## Abstract

**Objective:** This study aimed to report on perceived stigma by PLWH and factors that affect it.

**Materials and methods:** A cross-sectional study was carried out on 120 PLWH in Qom, Iran from November 2015 to April 2016. Persian version of Fife and Wright's scale was used to measure perceived stigma.

**Results:** The mean score of stigma was 73.19 ± 12.23 (range: 48-97). The means of external stigma and internal stigma were 43.70 ± 8.61 (range: 19-60), and 29.49 ± 5.32 (range: 17-40), respectively. Living in a rural areas (β = 10.341, p = 0.006), unemployment status (β = 6.910, p = 0.006), and poor support from family members (β = 4.772, p = 0.028) significantly increased the level of perceived stigma. PLWH experience a considerable level of stigma in their daily life. Mass media involvement to increase public awareness and reduce HIV related stigma need be more highlighted.

**Conclusion:** Working with the patients' families, interventions in the rural areas and workplaces, and providing social supports is necessary to minimize the HIV related stigma.

## Introduction

Based on World Health Organization (WHO) (1) estimations, about 0.8% of 15-49 year olds are living with HIV, worldwide. Based on a published governmental report, in Iran there were 28663 people living with HIV (PLWH) in 2014. However, the total number of PLWH has been estimated at 75700 persons (49600-135400, at CI: 95%). The prevalence of HIV infection in 15-49 year old adults was estimated at 0.14% in 2014, and this figure is expected to increase to 0.16% by 2020 (2). Although numerous efforts have been made to improve the rate of identification of PLWH and quality and accessibility of healthcare, there are many social, economic, cultural, and health system related barriers that impede process. Stigma related to HIV/AIDS is one of the most highlighted barriers in this regards (3, 4).

With the introduction of a combination of antiretroviral therapy (ART), HIV has become a chronic disease (5). PLWH face issues related to ageing, lifestyle changes, and chronic exposure to ART; therefore, the quality of life of these patients is an important issue (6-8). 

Perceived stigma greatly affects the quality of life of PLWH, their family members and the healthcare providers who work with them (9). Stigma causes serious care limitations for PLWH and this is an important factor for the HIV epidemic throughout the world (9-11). Shame, loss of self-efficacy, low self-esteem, low self-confidence and hopelessness associated with perceived stigma combined with side effects from ART, lead to poor adherence to ART, treatment failure, and the emergence of drug-resistant HIV strains (12-15).

Many factors affect PLWH’s perception of stigma and the influencing factors vary in different cultures, religion and geographical regions. Previous studies have reported gender, age and background factors, social class, social support, level of stereotypes, and the HIV transmission route as affecting factors on perceived stigma by PLWH (16-18). HIV related stigma is a result of complex interactions among social, contextual and self-factors. Accessibility of healthcare, social support, availability of appropriate information and economic, cultural, and political issues are some of the social factors that affect perceived stigma by PLWH. In contextual factors category, life conditions, drug and/or alcohol use, health status, time since diagnosis, and family circumstances are highlighted more in this regard. In the self-factors category, the presence of depression and anxiety, spirituality and meaning systems, coping skills, level of education, life skills and self-esteem are the most influencing factors (18, 19).

Few studies have been conducted on HIV related stigma in Iran, and these few available studies have not considered various aspects of stigma and factors associated with perceived stigma (20, 21). Karamouzian et al. in a qualitative study, tried to understand perceived stigma in PLWH. The majority of particiapants reported silence, shame and feeling miserable, which are types of internalized stigma. Participants also experienced stigma from their family members and healthcare providers (20). In SeyedAlinaghi et al.’s study 62% and 99% of participants experienced external and internal stigma, respectively (21). However, identifying affecting factors on perceived stigma was not the aim of their study. 

Although, some studies in Iran tried to report the perceived stigma by Iranian HIV infected persons but there are limited knowledge about factors associated with perceived stigma by Iranian HIV infected persons. Also, this study tried to explore the HIV related stigma and associated factors from a religious city in Iran which can be helpful to have a better understanding of perceived stigma by PLWH in a religious society. This study aimed to report the perceived HIV-related stigma and the affecting factors among PLWH in Qom, Iran.

## Materials and methods

***Setting and sample: ***A descriptive analytical cross-sectional study was conducted on 120 PLWH who presented at the consulting clinic of behavioral disorders in Qom, Iran from November 2015 to April 2016. The clinic is the only clinic that all PLWH in Qom are referred to in order to receive free healthcare. In this study, we included PLWH who were at least 18 years old, diagnosed at least one month before our study started and receiving ART at the clinic. We did not include PLWH who were suffering from any major psychological condition such as major depression and schizophrenia based on their medical documents at the clinic. We used the convenience sampling method with consideration to limit access to PLWH. We included all patients who attend the clinic during our study period. 

***Ethical consideration: ***Ethical approval was obtained from the Ethics Committee of the Qom University of Medical Sciences (NO: IR.MUQ.REC.1394.91). Informed consent was obtained from each of the participants. The questionnaires were anonymous and participation was voluntary.

***Measurement: ***We collected data including age, sex, marital status, place of residence, education level, job, the length of time since HIV diagnosis, and supposed route of transmission. We also asked participants to categorize their family members’ support in four categories (*very good to very bad*). 

We used Fife and Wright's (22) scale that was later revised by Kang et al. to measure perceived stigma related to HIV (23). Kang et al. reported a Cronbach’s alpha 0.9 for their revised scale (23). We received the scale developers' permission to translate and reprint the scale. This questionnaire was translated into Persian and its validity and reliability was evaluated. For the Persian version, Cronbach’s alpha coefficient was 0.85 for the overall scale (24). The scale consists of 20 items that measures internal and external stigma in five subscales including social rejection (9 items), negative self-worth (4 items), perceived interpersonal insecurity (2 items), financial insecurity (3 items), and discretionary disclosure (2 items).

External stigma was assessed based on the score of social rejection and financial insecurity subscales that represent perceived discrimination at their work place and in society and its consequences. Internal stigma was assessed based on the score of negative self-worth, perceived interpersonal insecurity, and discretionary disclosure represented subscales. Internal stigma indicates the extent to which the perceived discrimination and its consequences has been internalized and includes feelings of being apart from society, blaming oneself for the illness, and feeling a need to keep the illness a secret (23). Participants reported the extent of their agreement with each item based on a 5-degree Likert scale (1 = *strongly disagree*, 2 = *disagree*, 3 = *neutral*, 4 = *agree*, and 5 = *strongly agree*). With this scale, participants could receive a maximum score of 60 in external stigma, 40 in internal stigma, and 100 totally. A higher score indicated a stronger perceived stigma (24).

***Statistical analysis: ***All statistical analyses were done using IBM SPSS software version 21.0. Data were reported as mean ± standard or frequency counts and percentages. Independent *t* test, one way analysis of variance (ANOVA), and LSD post hoc test were used to compare means among groups. Also, to determine factors that affect HIV related stigma we performed stepwise multiple linear regressions. p < 0.05 was considered statistically significant.

## Results

***Participants’ demographic and HIV Characteristics:*** The mean age of the participants was 33.69 ± 7.86 (range: 19-55). The majority of them were men (63.6%) and resided in urban areas (91.1%). About 47% of the participants were never married, and 49.2% had less than a high school education. Twenty-five percent of the subjects were jobless. The most common route of HIV acquisition (45%) was sexual contact. The majority of the participants (62.5%) mentioned that their HIV was diagnosed during the current year. A high percentage (43%) rated their family support as bad (Table 1).

***Perceived Stigma: ***The mean score of stigma was 73.19 ± 12.23 (range: 48-97). The means of external and internal stigma were 43.70 ± 8.61 (range: 19-60) and 29.49 ± 5.32 (range: 17-40), respectively.

Mean scores for social rejection, financial insecurity subscale, negative self-worth subscale, perceived interpersonal insecurity, discretionary disclosure subscales were 33.75 ± 6.90 (range: 12-45), 13.28 ± 3.76 (range: 4-20), 7.99 ± 1.89 (range: 2-10), 9.94 ± 3.02 (range: 3-15), and 8.22 ± 1.91 (range: 2-10), respectively (Table 2).

**Table 1:** Demographic and disease related characteristics of participants

**Variables**		**n (%)**
Mean of age (year)		33.69 ± 7.86
Gender	Male	75 (63.6)
	Female	43 (36.4)
Marital status	Married	35 (31.3)
	Never married	53 (47.3)
	Divorced or widowed	24 (21.4)
Place of residence	Urban	102 (91.1)
	Rural	10 (8.9)
Educational level	Less than high school diploma	59 (49.2)
	High school diploma	48 (40.0)
	University	13 (10.8)
Job	Unemployed	30 (25)
	Worker or employee	27 (22.5)
	Self employed	41 (34.2)
	Housewife	22 (18.3)
Time post-HIV diagnosis (year)	˂ 1	75 (62.5)
	1-4	42 (35.0)
	≥ 4	3 (2.5)
Route of infection	Sharing needles	46 (38.3)
	Sexual contact	54 (45.0)
	Receiving blood	5 (4.2)
	Others	15 (12.5)
Family members’ support	Very good	12 (10.5)
	Good	39 (34.2)
	Bad	49 (43.0)
	Very bad	14 (12.3)

**Table 2:** Items and scale descriptive statistics on measures of perceived stigma (n = 120)

**Items**	**Strongly ** **Disagree ** **N (%)**	**Disagree ** **N (%)**	**Neutral ** **N (%)**	**Agree** **N (%)**	**Strongly** **Agree ** **N (%)**		**Mean** **Score ± SD**
Total score of stigma scale						73.19 ± 12.23	
*Social rejection (maximum score: 45)*							33.75 ± 6.90
1. My employer/coworkers have discriminated against me because of my illness	32 (26.7)	4 (3.3)	34 (28.3)	22 (18.3)	28 (23.3)		3.08 ± 1.49
2. Some people act as though I am less competent than usual	26 (21.7)	10 (8.3)	26 (21.7)	36 (30.0)	22 (18.3)		3.15 ± 1.41
3. I feel that I have been treated with less respect than usual by others	9 (7.5)	11 (9.2)	18 (15.0)	51 (42.5)	31 (25.8)		3.70 ± 1.17
4. I feel others are concerned they could ‘‘catch’’ my illness through contact like a handshake or eating food I make	11 (9.2)	3 (2.5)	14 (11.7)	46 (38.3)	46 (38.3)		3.94 ± 1.20
5. I feel others avoid me because of my illness	7 (5.8)	2 (1.7)	11 (9.2)	57 (47.5)	43 (35.8)		4.06 ± 1.02
6. Some family members have rejected me because of my illness	15 (12.5)	12 (10.0)	27 (22.5)	31 (25.8)	35 (29.2)		3.49 ± 1.34
7. I encounter embarrassing situations as a result of my illness	7 (5.8)	5 (4.2)	11 (9.2)	42 (35.0)	55 (45.8)		4.11 ± 1.11
8. I feel some friends have rejected me because of my illness	5 (4.2)	5 (4.2)	8 (6.7)	56 (46.7)	46 (38.3)		4.11 ± 0.99
9. Due to my illness, others seem to feel awkward and tense when they are around me	6 (5.0)	4 (3.3)	9 (7.5)	52 (43.3)	49 (40.8)		4.12 ± 1.03
*Negative self-worth (maximum score: 20)*							13.28 ± 3.76
10. I feel I am at least partially to blame for my illness	11 (9.2)	13 (10.8)	25 (20.8)	33 (27.5)	38 (31.7)		3.62 ± 1.28
11. I feel less competent than I did before my illness	22 (18.3)	11 (9.2)	28 (23.3)	43 (35.8)	16 (13.3)		3.17 ± 1.31
12. Due to my illness, I sometimes feel useless	12 (10.0)	11 (9.2)	19 (15.8)	52 (43.3)	26 (21.7)		3.58 ± 1.21
13. Changes in my appearance have affected my social relationships	24 (20.0)	24 (20.0)	23 (19.2)	35 (29.2)	14 (11.7)		2.92 ± 1.33
*Perceived interpersonal insecurity* *(maximum score: 10)*							7.99 ± 1.89
14. I feel I need to keep my illness a secret	5 (4.2)	13 (10.8)	16 (13.3)	40 (33.3)	46 (38.3)		3.91 ± 1.15
15. I have a greater need than usual for reassurance that others care about me	7 (5.8)	5 (4.2)	10 (8.3)	47 (39.2)	51 (42.5)		4.08 ± 1.10
*Financial insecurity (maximum score: 15)*							9.94 ± 3.02
16. I have experienced financial hardship that has affected how I feel about myself	13 (10.8)	14 (11.7)	24 (20.0)	41 (34.2)	28 (23.3)		3.48 ± 1.27
17. My job security has been affected by my illness	22 (18.3)	11 (9.2)	34 (28.3)	35 (29.2)	18 (15.0)		3.13 ± 1.31
18. I have experienced financial hardship that has affected my relationships with others	17 (14.2)	15 (12.5)	29 (24.2)	29 (24.2)	30 (25.0)		3.33 ± 1.36
*Discretionary disclosure (maximum score: 10)*							8.22 ± 1.91
19. I do not feel I can be open with others about my illness	3 (2.5)	15 (12.5)	12 (10.0)	39 (32.5)	51 (42.5)		4.00 ± 1.12
20. I fear someone telling others about my illness without my Permission	4 (3.3)	7 (5.8)	9 (7.5)	39 (32.5)	61 (50.8)		4.22 ± 1.04

**Table 3:** Comparison of means of perceived stigma related to HIV among different demographic variables

**Items**		**Mean score ± SD**	**Value of ** **T-test or F-test**	**p-value**
Gender	Male	72.27 ± 13.22	-1.061	0.291
	Female	74.77 ± 10.54		
Marital status	Married	74.89 ± 10.13	0.936	0.395
	Never married	71.32 ± 13.62		
	Divorced or widowed	71.87 ± 11.93		
Place of residence	Urban	72.08 ± 11.96	-2.602	0.011
	Rural	82.20 ± 8.68		
Educational level	Less than high school diploma	75.19 ± 11.72	2.624	0.077
	High school diploma	70.10 ± 12.48		
	University	75.54 ± 12.05		
Job	Unemployed	77.97 ± 12.02	4.336	0.006
	Employee	75.78 ± 14.08		
	Self-employed	68.45 ± 11.39		
	Housewife	72.27 ± 8.37		
Time post-HIV diagnosis (year)	˂ 1	71.83 ± 12.34	1.591	0.208
	1-4	75.07 ± 12.01		
	≥ 4	81.00 ± 5.00		
Route of infection	Sharing needles	74.50 ± 14.12	1.357	0.261
	Sexual contact	71.20 ± 10.87		
	Others	75.55 ± 10.66		
Family members’ support	Very good or good	69.69 ± 9.16	-3.045	0.003
	Bad or very bad	76.06 ± 13.67		

***Factors Associated with Stigma: ***In the bivariate analysis, the score of perceived stigma in patients who were residents of rural areas was significantly higher than urban residents (p = 0.011). Patients who were self-employed had significantly lower stigma scores than those who were unemployed (p = 0.001) or employees (p = 0.014). Patients who mentioned their family members’ support as bad or very bad perceived higher stigma than others (p = 0.003) (Table 3).

In linear logistic analysis, we included all variables that were statistically significant in the bivariate analysis. Based on linear regression analysis, being unemployed in comparison with self-employed (p = 0.006), receiving bad or very bad support from family members (p = 0.028), and residence in rural areas (p = 0.006) significantly increased the score of perceived stigma related to HIV (Table 4).

## Discussion

Based on our findings, PLWH in Iran face significant stigma and discrimination. The level of self-reported stigma was 73.19 ± 12.23 that was somewhat higher than reported levels in other countries using the same scale (22, 23, 25). Our participants perceived higher level of internal and external stigma than studies conducted in the USA (22, 23). In comparison with a study conducted in China, our participants perceived somewhat higher internal stigma but similar external stigma (25).

**Table 4:** Regression analysis of factors affecting perceived stigma score

**Variables**		**β coefficients**	**95% confidence interval**	**p-value**
Job	Self employed	Reference		
	Unemployed	6.910	2.067-11.752	0.006
Family members’ support	Very good or good	Reference		
	Bad or very bad	4.772	0.537-9.007	0.028
Place of residence	Urban	Reference		
	Rural	10.341	3.044-17.638	0.006

In our study scores of social rejection, perceived interpersonal insecurity and discretionary disclosure subscales were higher than other studies (22, 23, 25) However, in negative self-worth and financial insecurity subscales our participants reported similar levels of stigma with Fife et al. and Kang et al.'s studies (22, 23) and a lower level than Li et al.'s study (25). In previous studies in Iran, different methods and instruments were used; however, consistent with our findings, they reported a considerable level of stigma experienced by PLWH (20, 21). In Karamouzian et al.’s study the majority of particiapants reported silence, shame and feeling miserable, which are types of internalized stigma. Participants also experienced stigma from their family members and healthcare providers. However, identifying affecting factors on perceived stigma was not the aim of their study (20). SeyedAlinaghi et al. in a descriptive cross-sectional study done on a group of Iranian HIV positive patients evaluated a stigma index and reported on a percentage of people who preceived stigma from different people in different settings. Based on their findings, 62% of participants experienced external stigma and 99% reported internal stigma. They reported PLWH have limited access to jobs, education, and health services (21).

External stigma was assessed based on the score of social rejection and financial insecurity subscales that represent perceived discrimination at their work place and in society and its consequences. Internal stigma was assessed based on the score of negative self-worth, perceived interpersonal insecurity, and discretionary disclosure represented subscales. Internal stigma indicates the extent to which the perceived discrimination and its consequences has been internalized and includes feelings of being apart from society, blaming oneself for the illness, and feeling a need to keep the illness a secret (23).

The higher level of internal and external stigma in this study compared to studies in other countries may be due to awareness of HIV in those communities resulting in the acceptance of PLWH compared to Iran (21). Sexual contact and injecting drug use is the main route of HIV transmission worldwide as well as in Iran. Both sexual contact outside the marital framework and drug use in the Iranian religious society already carry their own stigmas, and certainly the combination of these situations strengthen the perceived stigma by PLWH (20). The finding that social stigma, related to the assumption that PLWH became infected through sexual activities, adultery, drug use and other morally dubious acts is evident (26, 27).

In Iran, the discussion of puberty, sexual health, and sexually transmitted diseases in families is limited and people do not receive appropriate information about them (28). HIV patients have always been concerned about the reactions of society. Sharing one’s HIV positive status causes "moral stigma" and the exclusion of patients from family, relatives and society. Also, the situation is much more severe in rural areas (29).

Based on our findings people living in rural areas compared to urban areas experienced more stigma. Heckman et al. mentioned rural HIV infected people compared with their urban counterparts, reported greater social stigma and less social support from family members, and lower levels of life satisfaction (30). Gonzalez et al. showed HIV infected women who are living in rural areas have more disclosure concerns than HIV infected women who are living in urban areas (31). Living in rural areas affects the accessibility and acceptability of HIV testing and healthcare, which increases mortality, morbidity and HIV transmission risk (32-34). Klichman et al. showed that AIDS related stigma is related to population density. People who are living in rural areas and towns experience greater internalized AIDS related stigma than people in large urban areas (35). In Teklehaimanot et al.'s study, people with farming occupations in rural areas are less likely to undergo voluntary HIV counseling and testing (36). Hence, PLWH who live in rural areas may be alone or isolated as one of the few people with the disease in their community. It is also possible that there is access to fewer resources than their urban counterparts due to less awareness about the disease and fear of disease transmission (30, 37, 38). Disclosure of the disease outside the family occurs more often in rural areas because it is difficult to hide secrets due to the small rural community and poverty that makes patients disclose their disease to receive public and subsidized services (39).

Another factor affecting the level of perceived stigma based on our findings was family support, such that those who feel more satisfaction with support of their family reported fewer stigmas. This finding is consistent with the findings of other studies (25, 37). Family support is likely to reduce internalized stigma and have a positive effect on patients’ self-esteem (25). A positive view of family members is important when PLWH disclose their status. Studies have shown that family support is crucial for PLWH to cope with the stress of HIV/AIDS and improve their quality of life. Lack of family support leads to experiencing stigma from family members and makes PLWH more vulnerable to HIV/AIDS related stigma in the society (25, 40, 41). Across all areas studied by Kalichman et al. internal stigma showed an inverse relationship with social support (42). Karamouzian et al. in their study in Iran reported HIV infected patients tried to hide their illness to protect family honor. The patients believed that their family honor and family support network would be damaged by disclosure of their status (20). In Iran, community is based on familial structures and peoples' experiences affect all their family members (43). Winskell et al. recognized that family members needed further education about HIV related issues, including stigma, disclosure, mental health, and responsibility for sexual relations (Winskell, Miller, Allen, & Obong'o, 2016). 

Another factor that showed a significant effect on perceived stigma was unemployment status. Perhaps perceived stigma by participants in their previous workplace led to unemployment or unemployment itself increased perceived stigma from different sources. However, in this study we did not investigate this issue. In addition, because self-employed patients perceived lower levels of stigma than the other work status group, this can be related to the circumstance of workplace. Also, those who are self-employed have more authority in their working environment and are not concern about disclosing illness at their workplace, and being fired. In the Li et al. study, farmers and the unemployed experienced more stigma than the self-employed (25). Stewart et al. showed employees suffer from the HIV stigma by colleagues and supervisors (39). Other studies also reported that PLWH experience stigma from colleagues and bosses in their workplace (42, 44). Rao et al. in their study showed that employers have a negative attitude toward hiring PLWH (14). Unemployed PLWH experience more mental health problems such as depression, suicidal thoughts and low self-esteem (45, 46). It is evident that unemployment carries its own stigma in the community and when PLWH became unemployed, the situation worsens. Furthermore, unemployed PLWH have to be dependent on other people or organisations that may increase the likelihood of experiencing stigma (47, 48).

***Limitations:*** This study was carried out in one city of Iran and may not be representative of all PLWH in Iran. The participants were approached at a health service center and were receiving ART, so we did not include those who were in the early stages of the disease and have not yet accessed HIV care. In this study because of limited access of PLWH, we had to apply the convenience sampling method. The participants psychological status was not assessed before including to the study as ART especially in starting period can have some psychological adverse effects our finding may be affected by this issue.

## Conclusion

This study is from a religious city in Iran and findings can improve our understanding of perceived stigma by PLWH in a religious environment. PLWH in our study perceived a considerable level of stigma in their daily life from different sources. Living in rural areas, poor support from family members, and unemployment significantly increase the perceived stigma.

Doing interventions to improve PLWH coping skills and flexibility can be effective to reduce the consequences of perceived stigma. Working with the patients' families and implementing interventions in the workplaces and providing other social support programs, as well as more attention to rural population is necessary to overcome the stigma related to HIV.

People should be provided with information that dispels myths related to HIV transmission risk in the home and workplace. Mass media involvement to increase public awareness and reduce HIV related stigma need be more highlighted. Use of mobile social networks, which are popular in Iran, can be helpful to provide educational programs for a broad range of the population. Involving religious leaders to improve the public's attitude can be effective to avoid moral judgments, prejudices, and stigmatization of PLWH. 

The knowledge and attitude of the employers toward HIV/AIDS should be assessed and based on the results; educational programs should be done for employers to improve their knowledge and attracting their support to destigmatize PLWH. Interventions to reduce stigma in hiring practices should be performed, as well. Providing some incentives for employers who hire PLWH can reduce the unemployment status of PLWH, which can contribute to the reduction of perceived stigma and better mental health. Providing financial and social support for patients’ families and engaging them in destigmatizing HIV/AIDS programs and improving their knowledge about HVI/AIDS can be helpful to eliminate HIV/AIDS related stigma as well. Existing rural health facilities in Iran provides an opportunity to increase rural population awareness, focusing on reduction of HIV related stigma. 

Studies are needed to find out appropriate and effective intervention considering the Iran social-cultural circumstances. Interventional studies need to be carried out on PWLH, their family and societies to assess the effect of different interventions on perceived stigma by PWLH.
